# Computer-navigated versus conventional total knee arthroplasty: A randomized controlled trial protocol in China

**DOI:** 10.1097/MD.0000000000021508

**Published:** 2020-08-07

**Authors:** Yefeng Yu, Jianming Sheng, Xiao Zhou

**Affiliations:** Department of orthopedics, Jiaxing Second Hospital, Zhejiang Province, China.

**Keywords:** computer-navigated, functional outcomes, study protocol, total knee arthroplasty

## Abstract

**Background:**

The literature lacks studies that confirm whether the improved radiographic alignment that can be achieved with computer-navigated total knee arthroplasty (TKA) improves patients’ activities of daily living or the durability of total knee prostheses. Thus, in this protocol, we designed a randomized controlled trial to compare implant alignment, functional scores, and survival of the implant using computer-assisted surgery versus a conventional surgical technique.

**Methods:**

This prospective, blinded randomized controlled trial was conducted at our single hospital. This study was approved by the ethics committee of Jiaxing Second Hospital. The patient inclusion criteria were age 20 to 80 years’ old, a body mass index of ≤35 kg/m^2^, and consented for primary knee arthroplasty performed through a medial parapatellar approach by the senior author. We randomized consented study participants on a 1:1 ratio to 1 of 2 study groups using a computer-generated list of random numbers in varying block sizes. The primary outcome in this study was the Knee Injury and Osteoarthritis Outcome Score. Secondary outcomes were the Knee Society Score, Western Ontario and McMaster Universities Osteoarthritis Index, complications, and range of motion together with alignment and rotational positioning of the implant. Statistical significance was defined as a *P* value of ≤0.05.

**Conclusions:**

Authors hypothesized that computer-assisted surgery in primary TKA improves implant alignment, functional scores, and survival of the implant compared to the conventional technique.

## 1 Introduction

Total knee arthroplasty (TKA) has emerged as one of the most successful treatment in orthopedic surgery, providing good long-term results with regard to pain reduction, functional improvement, and overall patient satisfaction.^[1]^ Nonetheless, up to 20% of patients continue to have pain or other knee symptoms after TKA. These symptoms can be related to postoperative malalignment of the knee.^[2]^ With the advent of computer-assisted surgery, accurate placement of the implant to within 0.5 to 1 mm is theoretically possible. Minor implant malpositioning can lead to early wear and loosening, thus worsening function and early failure. Computer-assisted surgery in TKA was developed to improve implant positioning and restore a neutral mechanical axis with the aim of improving patient function and implant survivorship.^[3–5]^

Improper alignment and imbalance can lead to increased and early polyethylene wear, early loosening, and decreased survival of implants.^[6]^ Advocates of computer-navigated TKA suggest that improved placement of the total knee components will lead to better midterm and long-term function and survival.^[7,8]^ However, clinical outcomes after navigation-assisted TKA have been found to be statistically similar to conventionally performed TKA at 2- or 5-year (short- and midterm) follow-ups.^[9–12]^ One meta-analysis of randomized controlled trials concluded that computer navigation significantly improved the mechanical axis of the leg and component positioning in TKA.^[13]^ Another meta-analysis found fewer outliers in the mechanical axis of the leg with computer navigation compared with conventionally aligned TKA, but the difference was not significant.^[14]^ Therefore, the role of computer navigation in TKA continues to be debated.

To our knowledge, in the literature, there are limited mid-term prospective randomized studies capable of answering to these questions. Thus, in this protocol, we designed a randomized controlled trial to compare implant alignment, functional scores and survival of the implant using computer-assisted surgery versus a conventional surgical technique. Authors hypothesized that computer-assisted surgery in primary TKA improves implant alignment, functional scores and survival of the implant compared to the conventional technique.

## 2 Material and method

### 2.1 Study design and patients

This prospective, blinded randomized controlled trial was conducted at our single hospital. This study was approved by the ethics committee of Jiaxing Second Hospital (HJX00903) and was registered in the Research Registry (researchregistry5757). All procedures were performed by a same senior surgeon and informed consent was obtained from each patient. Eighty eligible patients diagnosed at our institution with knee osteoarthritis during a period from July 2020 to July 2021 will be assessed.

The patient inclusion criteria were age 20 to 80 years, a body mass index of ≤35 kg/m^2^, and consented for primary knee arthroplasty performed through a medial parapatellar approach by the senior author. Exclusion criteria included an American Society of Anesthesiologists category of >3, which indicates severe systemic disease. Patients with severe neurological disease, dementia, previous cancer, a body mass index of >35 kg/m^2^, a previous tibial or femoral shaft fracture, severe preoperative valgus alignment of the knee (>15 degree from the mechanical leg axis), previous tibial or femoral osteotomy, recent knee injury (<1 year preoperatively), severe ipsilateral hip stiffness, ipsilateral hip replacement, or metal allergies were also excluded.

### 2.2 Randomization and blinding

**Figure 1:**
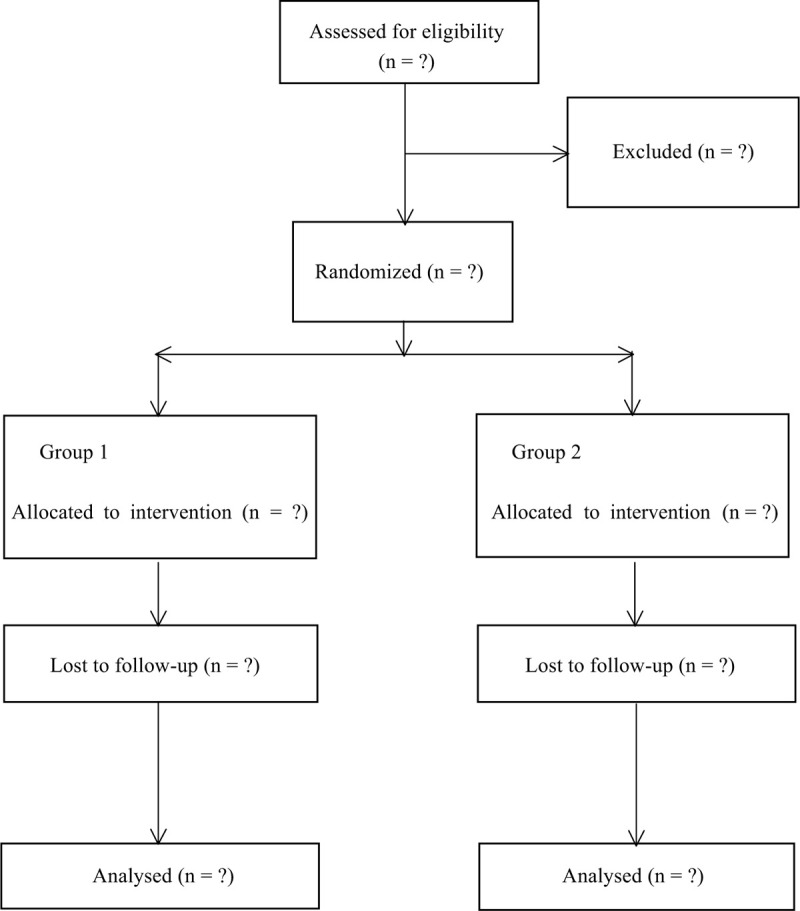
Consolidated Standards of Reporting Trials Statement flow diagram.

We randomized consented study participants on a 1:1 ratio to 1 of 2 study groups using a computer-generated list of random numbers in varying block sizes. An investigator with no further involvement in the study generated the allocation sequence using the Web site Randomization.com, and concealed the allocation results in sealed opaque sequentially numbered envelopes that were provided to the research coordinator. The surgeons, investigator, anesthetist, and nurses were all kept blinded to allocation results (Fig. 1).

### 2.3 Interventions

The conventional TKA was performed as described in the technique manual provided by the manufacturer (DePuy, Warsaw, IN). A mini medial parapatellar approach combined with an imageless computer navigation system (version 1.0; DePuy or Brainlab, Feld-kirchen, Germany) was used to perform the computer-navigated TKA. The midline skin incision was <10 cm long. An abbreviated quadriceps tendon-splitting approach was used, without patellar eversion. All components (femoral, tibial, and patellar) were cemented, and a tourniquet was used throughout the procedure in all cases. Perioperative pain management, prophylaxis against deep vein thrombosis, and physiotherapy were standardized.

### 2.4 Outcomes measures

The primary outcome in this study was the Knee Injury and Osteoarthritis Outcome Score (KOOS). The follow-up period was 5 years, with scheduled follow-up visits at 1, 3, and 5 years. KOOS is a 42-item self-administered questionnaire with 5 subscales: pain (9 items), other symptoms (7 items), activities of daily living (17 items), sport and recreational function (5 items), and knee-related quality of life (4 items). The minimal clinically important difference (MCID) for KOOS used on knee replacement patients has not been determined; however, a change in the KOOS pain score of 8 to 10 has been used earlier as a suggested MCID.

Secondary outcomes were the Knee Society Score (KSS), Western Ontario and McMaster Universities Osteoarthritis Index (WOMAC), complications, and range of motion together with alignment and rotational positioning of the implant. KSS functional scores were assessed preoperatively, and after 1, 3, and 5 years by physiotherapists. The WOMAC is a widely used, disease-specific measure for knee osteoarthritis with a multidimensional scale consisting of 24 items grouped into 3 dimensions: pain (5 items), stiffness (2 items), and physical function (17 items). WOMAC scores can be derived from the KOOS questionnaire.

### 2.5 Sample size calculation

To minimize the chance of type-2 error and increase the power of our study, we assumed the difference in the KOOS score to be 8 points with a power of 0.90, which revealed that a total of 40 patients would be needed in each group. We recruited approximately 10% more patients to account for possible dropouts. Intraobserver reliability was almost perfect for both the computer-navigated and the conventional TKAs.

### 2.6 Statistical analysis

Statistical analyses were carried out using SPSS 21.0 (IBM, Armonk, NY). Testing for normality was done with the Shapiroe-Wilk test. The Mann–Whitney *U* test was used for continuous variables with non-normal distribution, whereas the Student unpaired *t* test was used for continuous variables with normal distribution (age, body mass index, range of motion, and all component angels; standard deviation was reported). For categorical variables, the Pearson *χ*^2^ test was used. Statistical significance was defined as a *P* value of ≤0.05.

## 3 Discussion

Computer navigation technology is “designed to improve the surgical performance and clinical outcome of knee replacement surgery,” and with this in mind, its use has increased in recent years.^[15–20]^ In parallel, the volume of literature relating to computer navigation technology in this field has expanded greatly, most published work relating to component alignment, whereas very few studies have addressed functional outcome.^[21–24]^ Thus, in this protocol, we designed a randomized controlled trial to compare implant alignment, functional scores, and survival of the implant using computer-assisted surgery versus a conventional surgical technique. Authors hypothesized that computer-assisted surgery in primary TKA improves implant alignment, functional scores, and survival of the implant compared to the conventional technique.

## Author contributions

**Conceptualization:** Xiao Zhou.

**Data curation:** Yefeng Yu.

**Formal analysis:** Yefeng Yu, Jianming Sheng.

**Funding acquisition:** Xiao Zhou.

**Investigation:** Yefeng Yu, Jianming Sheng.

**Methodology:** Xiao Zhou.

**Resources:** Xiao Zhou.

**Software:** Jianming Sheng.

**Supervision:** Xiao Zhou.

**Validation:** Jianming Sheng.

**Visualization:** Jianming Sheng.

**Writing – original draft:** Yefeng Yu.

**Writing – review & editing:** Xiao Zhou.
